# Dentin Loss and Surface Alteration Through Chemical and Chemomechanical Challenge after Initial Root Instrumentation

**DOI:** 10.3290/j.ohpd.b4100941

**Published:** 2023-05-17

**Authors:** Adrian Christian Frey, Andrea Gubler, Patrick R. Schmidlin, Florian J. Wegehaupt

**Affiliations:** a Dental Master’s Student, Clinic of Preventive Dentistry, Periodontology and Cariology, Center of Dental Medicine, University of Zürich, Zürich, Switzerland. Idea of the present study, performed the experiments in partial fulfilment of requirements for a Master’s degree and wrote the manuscript.; b Lab Manager, Clinic of Preventive Dentistry, Periodontology and Cariology, Center of Dental Medicine, University of Zürich, Zürich, Switzerland. Experimental design, contributed substantially to discussion and writing the paper, proofread the manuscript.; c Professor and Head, Division of Periodontology and Peri-implant Diseases, Clinic of Conservative and Preventive Dentistry, Center for Dental Medicine, University of Zürich, Zürich, Switzerland. Experimental design, contributed substantially to discussion and writing the paper, proofread the manuscript.; d Assistant Professor and Head of Division of Preventive Dentistry and Oral Epidemiology, Clinic of Conservative and Preventive Dentistry, Center for Dental Medicine, University of Zürich, Zürich, Switzerland. Experimental design, contributed substantially to discussion and writing the paper, proofread the manuscript.

**Keywords:** erythritol airflow, hand scaler, ultrasonic, substance loss, surface roughness.

## Abstract

**Purpose:**

To assess the root surface roughness and substance loss induced by chemical and chemomechanical challenges on root surfaces pretreated with ultrasonic instrumentation, a hand scaler, or erythritol airflow.

**Materials and Methods:**

One hundred twenty (120) bovine dentin specimens were used in this study. Specimens were divided into eight groups and treated as follows: groups 1 and 2: polished with 2000- and 4000-grit carborundum papers but not instrumented (‘untreated’); groups 3 and 4: hand scaler; groups 5 and 6: ultrasonic instrumentation; groups 7 and 8: erythritol airflow. Samples from groups 1, 3, 5, and 7 then underwent a chemical challenge (5 x 2 min HCl [pH 2.7]), whereas samples from groups 2, 4, 6, and 8 were subjected to a chemomechanical challenge (5 x 2 min HCl [pH 2.7] + 2 min brushing). Surface roughness and substance loss were measured profilometrically.

**Results:**

The least substance loss through chemomechanical challenge was noted after erythritol airflow treatment (4.65 ± 0.93 µm), followed by ultrasonic instrumentation (7.30 ± 1.42 µm) and the hand scaler (8.30 ± 1.38 µm); the last two (hand scaler and ultrasonic tip) did not differ statistically significantly. The highest roughness after chemomechanical challenge was observed on ultrasonically treated specimens (1.25 ± 0.85 µm), followed by hand-scaled specimens (0.24 ± 0.16 µm) and those subject to erythritol airflow (0.18 ± 0.09 µm); there was no statistically signficant difference between the latter two, but they both differed statistically significantly from the ultrasonically treated specimens. No statistically significant difference in substance loss through the chemical challenge was observed between specimens pretreated by the hand scaler (0.75 ± 0.15 µm), ultrasonic tip (0.65 ± 0.15 µm), and erythritol airflow (0.75 ± 0.15 µm). The chemical challenge smoothed the surfaces treated with the hand scaler, ultrasonic tip, and erythritol airflow.

**Conclusion:**

Dentin pretreatment with erythritol powder airflow resulted in a higher resistance to chemomechanical challenge than did dentin treated ultrasonically or with the hand scaler.

The main clinical objectives of systematic periodontal therapy are the reduction of inflammation with absence of bleeding on probing, the reduction of pocket depths, and a increase/stabilisation of attachment levels.^15,27,31,34^ Un- or insufficiently treated, instable periodontal conditions can even lead to tooth loss.^12^ The effective removal of biofilm and calculus in the second step of periodontal therapy as cause-related therapy, i.e. the anti-infective treatment, is of central importance for successful treatment and secondary prevention.^34^

The balance between efficient removal of dental soft and hard depositions on the one hand and tooth substance loss as well as morphological alterations after root instrumentation on the other is critical in terms of the benefit and harm of periodontal therapy.^36^ Different studies evaluated the efficacy of a plethora of different instruments and determined no clinically or statistically significant difference between hand curette, ultrasonic or air polishing devices in terms of biofilm removal.^25,38^ Concerning the tooth substance loss, curettes led to the most pronounced tooth substance removal.^20,32^ On the other hand, surface roughness was highest after ultrasonic instrumentation, whereas the smoothest surfaces were obtained using hand instruments.^5,16,23,35,41^ However, other studies reported contrasting results, which depended on the pretreatment of the tooth samples, as well as different application modalities such as pressure, power setting, duration of application, etc.^8,9,26^

From a clinical perspective, diseased sites display either pre-existing exposed roots or recessions which occur due to shrinkage of the inflamed soft tissues. During individual patient care, repeated surface instrumentation makes the tooth surface less resistant to erosion and mechanical abrasion by the individual’s tooth cleaning measures at home.^1,2,40^ The additional substance loss, however, may greatly vary with the ingredients of the toothpaste; the structure of the brush^7^ and the micromorphology of the root surface may also play a distinctive role, especially after surface alterations during professional root-surface instrumentation.

According to the authors’ knowledge, little attention has been paid up to now to these characteristics immediately after root surface treatment. Therefore, it was the aim of the present study to compare the chemical and the combined chemomechanical dentin loss as well as the root surface micromorphology on dentin surfaces pretreated with various commonly used cleaning measures, i.e. hand scaler, ultrasonic tip and erythritol airflow technology.

We hypothesised that there was no difference in root surface roughness and tooth substance loss after chemical and chemomechanical challenge between the different root surfaces generated by a hand scaler, an ultrasonic tip and erythritol airflow technology.

## Materials and Methods

### Experimental Procedure

#### Sample preparation and standard surface treatment

One hundred twenty root samples were milled out of bovine mandibular incisors under constant water cooling, using a cylindrical diamond-coated trephine mill with an inner diameter of 5 mm. The samples were embedded in acrylic resin (Paladur, Heraeus Kulzer; Hanau, Germany) using a silicone template with an inner diameter of 1 cm. The acrylic resin was polymerized at 55°C and 2 bar for 10 min in a laboratory polymerization unit (Palamat elite, Heraeus Kulzer). In order to completely remove the cementum layer of the roots and create a standardised dentin surface among all samples, standardised polishing was performed in an automatic grinding machine using 2000- and 4000-grit carborundum papers (Tegramin-30, Struers; Copenhagen, Denmark) at a pressure of 1 N for 30 s under constant water cooling. As reference points for the subsequent profilometric measurements, two parallel notches on each side of the dentin sample were then made in the acrylic embedding material. To obtain and maintain a reference area for profilometric analysis, the dentin and acrylic resin of each specimen was protected with two pieces of adhesive tape during pretreatment, acid exposure and brushing cycles, leaving an unprotected area of around 15 mm^2^. The experimental design is summarised in Table 1.

**Table 1:** Experimental design

120 dentin samples from bovine roots (5 mm)							
Sample preparation and standard surface treatment							
Recording of baseline profiles (profilometer)							
Untreated control		Hand scaler (M23, Deppeler; Rolle, Switzerland)		Ultrasonic tip (PiezoLED 202 point, KaVo; Biberach, Germany)		Erythritol airflow (EMS; Nyon, Switzerland)	
Group 1 n = 15	Group 2 n = 15	Group 3 n = 15	Group 4 n = 15	Group 5 n = 15	Group 6 n = 15	Group 7 n = 15	Group 8 n = 15
Recording of profiles (profilometer) and scanning electron microscopy							
Chemical challenge: 5 x 2 min HCl (pH 2.7)	Chemomechanical challenge: 5x (2 min HCl + 2 min brushing)	Chemical challenge: 5 x 2 min HCl (pH 2.7)	Chemomechanical challenge: 5x (2 min HCl + 2 min brushing)	Chemical challenge: 5 x 2 min HCl (pH 2.7)	Chemomechanical challenge:5x (2 min HCl + 2 min brushing)	Chemical challenge: 5 x 2 min HCl (pH 2.7)	Chemomechanical challenge: 5x (2 min HCl + 2 min brushing)
Recording of final profiles (profilometer) and scanning electron microscopy							

#### Instrumentation

The dentin samples were then randomly allocated to the different groups. Groups 1 and 2 were left untreated (polished with 2000- and 4000-grit carborundum papers but not instrumented) and served as controls. Groups 3 and 4 were treated with a hand scaler (M23, Deppeler; Rolle, Switzerland) for 10 strokes and a pressure of 200 g. The specimens of groups 5 and 6 were treated with an ultrasonic tip (PiezoLED 202 point, KaVo; Biberach, Germany) for 20 s and 40 g on the high power setting. Groups 7 and 8 were treated with an airflow device (EMS; Nyon, Switzerland) on powder setting level 5 and water setting level 5 for 10 s with the erythritol powder at a working distance of 5 mm and an angle of 90 degrees. The instrumentation was performed by one operator.

#### Chemical challenge

Groups 1, 3, 5 and 7 were then exposed to a chemical challenge. Specimens were eroded for two min in HCl with a pH of 2.7. After erosion, the specimens were rinsed with tap water and then put into artificial saliva prepared following the formulation given by Klimek et al^19^ for 5 min. These cycles were repeated 5 times, so that every specimen was eroded for 10 min with HCl.

#### Chemomechanical challenge

Groups 2, 4, 6 and 8 were subjected to a chemomechanical challenge. Every two dentin samples were placed in a plastic brushing container. The samples were then eroded for 2 min with HCl with a pH of 2.7, then rinsed with tap water to stop the erosive attack. Afterwards, 6 brushing containers were screwed tight onto one of six places of the brushing machine (custom-made, Clinic of Conservative and Preventive Dentistry, Center of Dental Medicine, University of Zürich, Switzerland) and brushing was performed with a medium-hard standard toothbrush (Paro M43, Esro; Thalwil, Switzerland) for 2 min. The brushing frequency used was 60 strokes/min and the load applied by the toothbrush was set at 2 N. The slurry used for the brushing sequence was prepared with 75 g of Sident (Evonik Industries; Essen, Germany) ISO 2480-1 RDA 85, 0.375 g of Antifoam (Sigma-Aldrich; St Louis,MO,USA) and a saliva substitute containing HEC and glycerin (both Sigma-Aldrich), creating a slurry with an RDA of 80. Two milliliters of this slurry were added to each brushing container. After the brushing cycle, the specimens were rinsed with tap water again and were immersed in artificial saliva for 5 min. This sequence was repeated 5 times until a total brushing time of 10 min and 10 min of HCl exposure was reached.

### Measurement and Methodology

The measurements in this study were performed with two different contact profilometers (Perthometer S2, Mahr; Göttingen, Germany, and Talysurf Series 2, Taylor-Hobson; Leicester, England) for substance loss and surface roughness, respectively. Five parallel surface profiles with a distance of 500 µm and a recording accuracy of 40 nm for the Perthometer and 10 nm for the Talysurf were recorded for each sample. To maximize the accuracy of specimen repositioning in the profilometer, a prefabricated jig was used. Surface loss and roughness were recorded after specimen preparation, after instrumentation and finally after the respective challenges (chemical or chemomechanical). Dentin wear was then calculated with custom-made software which superimposed all the profiles recorded for each specimen. The profilometric analysis has been described in detail in a previous publication by Attin et al.^4^ Additionally, electron microscopy images from previously selected samples were taken after instrumentation and chemical/chemomechanical challenge. The images were taken with the Zeiss Gemini SEM 450 (Jena, Germany) at 10.00 kV and 200 pA.

### Statistical Analysis

Medians and interquartile ranges of dentin wear through instrumentation and chemical/chemomechanical challenges, as well as the surface roughness after standard surface treatment, instrumentation and chemical/chemomechanical challenges were calculated for every sample. Differences between the groups were then tested using Kruskal-Wallis Omnibus test (p<0.05). To determine whether the roughness changed through chemical/chemomechanical challenge, the difference of roughness, DRa (Ra after chemical/chemomechanical challenge – Ra after instrumentation), was tested against 0 with the Wilcoxon signed-rank test. The resulting p-values were then corrected for multiple testing according to Holm. All statistical analyses and plots were performed using statistical software (R version 4.2.1 ‘Funny-Looking Kid’ including the packages ggplot2 version 3.3.6, knitr version 1.41, and PMCMRplus version 1.9.6).^28,30,43,45^

## Results

### Substance Loss

Dentin loss due to mechanical instrumentation, chemical and chemomechanical challenge is presented in Table 2. The abrasive dentin wear resulting from instrumentation varied considerably between the different instruments. Samples treated with a hand scaler showed the highest amount (median [IQR]) of abrasive dentin wear (12.38 [3.44] µm), followed by erythritol airflow (4.70 [1.85] µm), while the least substance loss resulted from ultrasonic treatment (1.77 [0.73] µm). Wear due to instrumentation was statistically significantly different between all groups (p < 2e-16). The dentin samples that underwent chemical challenge showed no statistically significant differences in substance loss on the variously treated surfaces, i.e. untreated or by hand scaler, ultrasonic instrumentation, and erythritol airflow (untreated vs hand scaler: p = 0.07; untreated vs ultrasonic: p = 1.00; untreated vs erythritol airflow: p = 0.13; hand scaler vs ultrasonic: p = 0.07; hand scaler vs erythritol: p = 1.00; ultrasonic vs erythritol airflow: p = 0.13). On the other hand, the chemomechanical challenge yielded statistically significantly less substance loss on surfaces treated with erythritol airflow than on samples treated with a hand scaler, ultrasonic tip, or untreated surfaces (erythritol airflow vs untreated: p = 7.8e-07; erythritol airflow vs hand scaler: p = 3.4e-08; erythritol airflow vs ultrasonic: p = 1.9e-05). Between these groups, no statistically significant difference in dentin loss was observed (untreated vs hand scaler: p = 0.72; untreated vs ultrasonic: p = 0.72; hand scaler vs ultrasonic: p = 0.23).

**Table 2:** Median values and interquartile range (µm) of substance loss through instrumentation, chemical challenge or chemomechanical challenge

	Instrumentation	Chemical challenge	Chemomechanical challenge
Instrument			
Untreated	0 ± 0.00 A	0.70 ± 0.17 A	7.80 ± 1.88 A
Hand scaler	12.38 ± 3.44 B	0.75 ± 0.15 A	8.30 ± 1.38 A
Ultrasonic device	1.77 ± 0.73 C	0.65 ± 0.15 A	7.30 ± 1.42 A
Erythritol airflow	4.70 ± 1.85 D	0.75 ± 0.15 A	4.65 ± 0.93 B

The chemical and the chemomechanical challenges were executed on the surfaces generated through instrumentation. Values that are not statistically significantly different are marked with same capital letters (testing concerned only the different instruments at the same point of time [read vertically]).

### Median Ra Values

**Fig 1:**
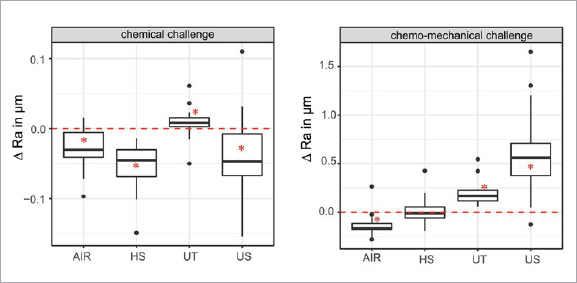
Boxplot with median and quartiles of roughness changes (△Ra = Ra after chemical/chemomechanical challenge - Ra after instrumentation) generated by the chemical and chemomechanical challenge on the different surfaces made by UT: untreated; HS: hand scaler; US: ultrasonic device; AIR: airflow (erythritol). The dotted red line marks 0, which means no changes in roughness due to chemical or chemomechanical challenge. Box plots marked with an asterisk (*) differ statistically signifcantly from 0.

Dentin roughness due to mechanical instrumentation, chemical and chemomechanical challenge is presented in Table 3. Dentin roughness changes due to chemical and chemomechanical challenge are presented in Fig 1. The median roughness values Ra plus interquartile range of the samples treated with the hand scaler (0.25 [0.17] µm) and erythritol airflow (0.30 [0.11] µm) did not differ statistically significantly (p = 0.8). In contrast, treatment with an ultrasonic tip (0.75 [0.45] µm) created a statistically significantly rougher surface compared to the hand scaler and erythritol airflow (ultrasonic vs hand scaler: p = 1.2e-09; ultrasonic vs erythritol airflow: p = 1.6e-09). The untreated dentin surface showed the statistically significantly lowest median Ra value (0.02 [0.01] µm) (untreated vs hand scaler: p = 1.2e-09; untreated vs ultrasonic device <2e-16; untreated vs erythritol airflow: p = 7.6e-10). The chemical challenge led to smoothening of the samples whose surfaces were treated with the hand scaler, ultrasonic tip and erythritol airflow. The surfaces of untreated samples were roughened by the chemical challenge. Only for the samples treated with the hand scaler and erythritol airflow did the values differ statistically significantly from 0 (Ra after chemical challenge - Ra after instrumentation) (untreated: p = 0.13; hand scaler: p = 4.9e-04; ultrasonic: p = 0.06; erythritol airflow: p = 0.04) (Fig 1). The chemomechanical challenge roughened the surface of untreated samples as well as samples treated with the ultrasonic tip. The differences (Ra after chemomechanical challenge - Ra after instrumentation) were both statistically significantly higher than 0 (untreated: p = 4.9e-04; ultrasonic: p = 1.1e-03). However, the difference in Ra value of the untreated samples increased to a lesser degree than did that of samples treated with the ultrasonic tip (Fig 1). No statistically significant changes in the Ra values through chemomechanical challenge were detected on the surface treated with the hand scaler (p = 0.95). The chemomechanical challenge smoothed the surface of samples treated with erythritol airflow. This change was statistically significant (p = 0.027) (Fig 1). The median Ra values plus interquartile range generated by the chemomechanical challenge did not differ statistically significantly between the samples treated with the hand scaler, erythritol airflow, and untreated samples (untreated vs hand scaler: p = 0.45; untreated vs erythritol airflow: p = 0.45; hand scaler vs erythritol airflow: p = 0.12). Only the median Ra value for the ultrasonically instrumented samples was statistically significantly higher after chemomechanical challenge than the median Ra after chemomechanical challenge of the samples treated with the hand scaler, erythritol airflow, and untreated samples (ultrasonic vs untreated: p = 6.7e-08; ultrasonic vs hand scaler: p = 5.3e-06; ultrasonic vs erythritol airflow: p = 2.7e-09).

**Fig 2:**
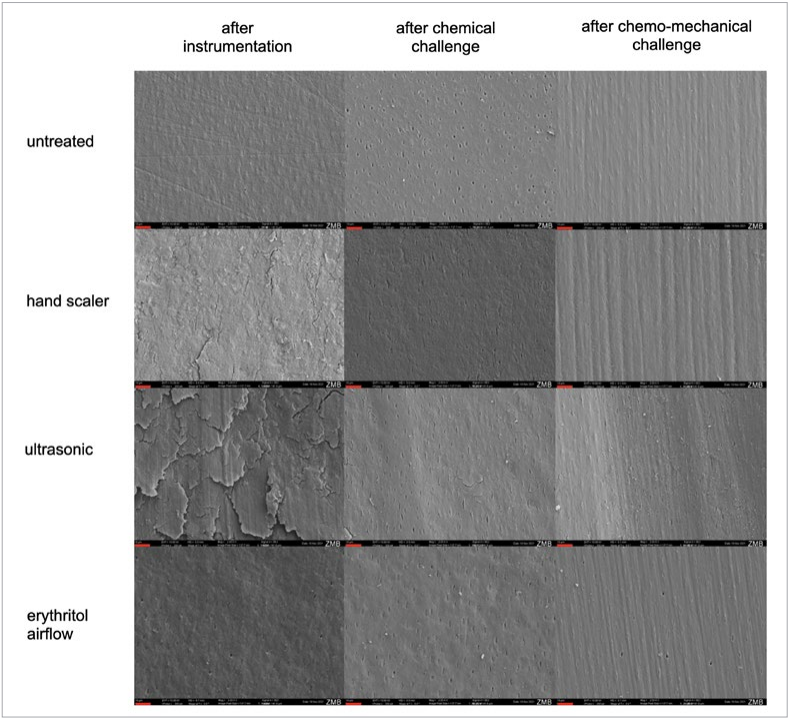
SEM images of dentin surfaces after instrumentation with the hand scaler, ultrasonic device, airflow, or left untreated, after chemical/chemomechanical challenge. The red line marks 10 µm.

**Table 3:** Median values and interquartile range in µm of surface roughness (Ra) before treatment, after instrumentation, after chemical or chemomechanical challenge

	Before instrumentation	After instrumentation	After chemical challenge	After chemomechanical challenge
Instrument				
Untreated	0.02 ± 0.01 A	0.02 ± 0.01 A	0.03 ± 0.01 A	0.20 ± 0.11 A
Hand scaler	0.02 ± 0.01 A	0.25 ± 0.17 B	0.21 ± 0.15 B	0.24 ± 0.16 A
Ultrasonic device	0.02 ± 0.01 A	0.75 ± 0.45 C	0.70 ± 0.47 C	1.25 ± 0.85 B
Erythritol airflow	0.02 ± 0.01 A	0.30 ± 0.11 B	0.20 ± 0.14 B	0.18 ± 0.09 A

The chemical and the chemomechanical challenges were performed on the variously instrumented surfaces. Values that are not statistically significantly different are marked with same capital letters (testing concerned only the different instruments at the same point of time [read vertically]).

### SEM Observations

The SEM images reflect the roughness values given in Table 3. After instrumentation with the hand scaler and erythritol airflow, the micrographs showed similar surface topographies. Erythritol airflow produced a more homogeneous surface, while the hand scaler created a few scale-like structures. After ultrasonic treatment, the surface appeared rougher and more irregular, with considerable chipping. The untreated specimen showed a smooth, polished surface with some thin scratches resulting from polishing. The chemical challenge led to smoothening of the samples, as illustrated by the complete removal of the scales produced by the hand scaler and partial removal of the scales from the ultrasonic samples. The surface of erythritol-airflow–treated samples after chemical challenge appears softer and shinier. Furthermore, the dentin tubules on every sample are more visible after chemical challenge compared to erythritol-airflow–treated samples, which still exhibited some obliterated tubules. The surfaces of untreated, hand-scaler–instrumented and erythritol-airflow–treated samples after chemomechanical challenge showed a very similar structure, with parallel notches resulting from the brushing cycles. The notches on the hand-scaler–treated samples were slightly wider and appeared a little deeper than the ones on the untreated and erythritol-airflow–treated specimens. The very deep, wide notches produced by the brush on the samples pretreated with the ultrasonic tip are conspicuous.

## Discussion

To maintain good oral hygiene and treat periodontitis, professional tooth cleaning or scaling plays a major role. The removal of dental hard tissue and changes in surface morphology inherent in this are well known. The main objective of this study was to determine what happens afterwards regarding surface roughness and substance loss on the treated rooth surfaces by toothbrushing at home.

To this end, a universal hand scaler (Deppeler, M23), an ultrasonic device (KaVo, PiezoLED) and an erythritol airflow (EMS) were compared regarding dentin removal and surface roughness changes on bovine root surfaces. In addition, the changes of roughness and substance removal by chemical and chemomechanical challenge were tested on surfaces treated with the three instruments. The roughness of the root following instrumentation is a much-discussed topic in dentistry. Mierau^24^ and Quirynen and Bollen^29^ showed that a rough root surface led to bacterial adhesion and further to plaque formation. The removal of dental hard tissue must be minimised; otherwise it can result in irreversible tooth damage, hypersensitivity and, in extreme cases, even vitality loss or tooth fractures.

The dentin samples in this study were harvested from bovine roots. Bovine dentin is a suitable substitute for human teeth when used in experiments investigating erosive and abrasive changes of the surface.^42^ Between the treatments and the measurements, the samples were stored in tap water, which proved to have no influence on the results.^4^ For a meaningful measurement of tooth substance loss as well as surface roughness, a profilometer was utilised.^13^

Flemmig et al^10^ have shown that substance loss and defect depth produced by piezoelectric and magnetostrictive ultrasonic instruments are determined by instrumentation time, lateral forces, power setting and tip angulation. Consequently, the safest tip angulation of 0° and the maximum power setting were chosen for the PiezoLED ultrasonic device.

A study by Ritz et al^33^ demonstrated that for the Titan-S scaler, oscillation decreases linearly with prolonged application force. With a maximum amplitude of 140 µm at 0 g, decreasing to 80 g where no oscillation was detected, a force of 40 g for the ultrasonic device was chosen for this study.

A problem with in-vitro research concerning the use of hand scaler is the heterogeneity and the lack of standardisation in application pressure. Canakci et al^6^ showed that the mean application force in vivo is 0.93 N with a non-pen grip. In the in-vitro analysis by Schmidlin et al,^36^ a pressure of 500 g was applied. Other studies simply reported an appropriate amount of pressure during the strokes.^46^ Given this background, a pressure of 200 g was chosen for the use of the hand scaler.

Kröger et al^21^ demonstrated that the application angle and the different pressure settings of the airflow device with erythritol powder have a minor influence on substance loss. Only the distance of the nozzle to the treated surface plays a major role. Those authors investigated the substance loss at three different distances: 1, 3, and 5 mm. For our study we therefore chose the medium pressure setting with a working distance of 5 mm.

The duration of application and the number of strokes were chosen by the operators to treat the dentin surface, which was approximately 15 mm^2^, as in a clinical setting. The duration of acid exposition per cycle at 2 min as well as the storage of the samples in artificial saliva (see Klimek et al^19^) were chosen based on recommendations by Wiegand and Attin.^44^

In terms of brushing force in erosion/abrasion studies, Wiegand and Attin^44^ suggested standardising the value between 1 and 2 N. Therefore, a brushing load of 2 N was chosen for the present study. To create a standard setting, the standard medium toothbrush Paro M43 was used and a slurry with an RDA of 80 without fluoride was chosen as a toothpaste substitute for all samples. It is known that fluoridated toothpastes create less substance wear on eroded dentin in vitro.^14,22^ As our focus was on the influence of the different physical properties/surface morphologies, non-fluoridated toothpaste was used to eliminate the remineralising/anti-erosive effect of fluoride.^39^ 120 strokes per brushing cycle is at the upper limit, as more than 100 brushing strokes per cycle are considered extensive brushing and often result in a higher amount of dentin wear than in a clinical situation.^44^ Another limiting factor might be that most people do not brush their teeth after each acidic food or drink intake. The imbalance between erosion and abrasion resulting from this set-up was not considered. However, it is well known that the degree of abrasivity is higher on pre-eroded surfaces than on native surfaces.^3^

The results of the present study regarding instrumentation appear to have produced outcomes similar to those of previous studies which investigated surface roughness and substance loss by instrumentation with ultrasonic devices, hand scalers and erythritol airflow. This was important because the following steps (chemical challenge, chemomechanical challenge) of the experiment relied on a representative surface to provide meaningful results. As in most studies, the smoothest surface and the highest substance loss was detected for the hand scaler (substance loss: 12.38 ± 3.44 µm, Ra: 0.25 ± 0.17 µm), and the highest surface roughness and the least substance loss was found for the ultrasonic device (substance loss: 1.77 ± 0.73 µm, Ra: 0.75 ± 0.45 µm).^16,17,23,36,37,41^ It is difficult to compare the actual values since there is no standardisation between different studies for the use of hand scalers and ultrasonic devices. The present results for erythritol airflow showed a substance loss which was lower than that produced by the hand scaler but greater than that created by the ultrasonic device (substance loss: 4.70 ± 1.85 µm). It is noteworthy that with the same settings (pressure and distance), Kröger et al^21^ found a higher amount of substance loss (18 ± 7 µm). The explanation for this discrepancy is that instrumentation was statically fixed and not moved in the study by Kröger et al.^21^ In the present study, the handpiece was moved over the 15 mm^2^ test surface to treat the whole surface regularly, as with the hand scaler and ultrasonic device.

Analysing the results of the chemical challenge, the null hypothesis regarding substance loss could be confirmed. No difference of substance loss through chemical challenge on the different surfaces produced by the hand scaler, ultrasonic tip, erythritol airflow and the untreated surface was observed. Ganss et al^11^ demonstrated deeper erosions on polished root surface samples than on untreated, rougher surfaces. These findings contradict the present results. It may be assumed that these dissimilar results were obtained through different chemical challenges. Ganss et al^11^ treated their samples with citric acid for 3 h at 37°C, whereas the samples in the present study were treated 5 times for 2 min each (total: 10 min) at room temperature with HCl (pH 2.7). On the other hand, the hypothesis that the changes in roughness through chemical challenge do not differ between the different surface morphologies had to be rejected. Smoothing via chemical challenge was observed on the samples treated with the hand scaler, ultrasonic tip and erythritol airflow. However, roughening of the surfaces not treated with an instrument but instead only with standardised polishing using 2000- and 4000-grit carborundum papers was observed. Based on these findings, one might assume that on a macroscopic scale, the acid attack is irregular on uneven surfaces and starts at the surface spikes, thus creating a smoother surface. However, if a surface is completely flat, the erosive attack starts evenly on a macroscopic but not a microscopic scale. Initially the erosive attack starts at similar rates in the peri- and intertubular dentin; after a few minutes, the intertubular region is less demineralised than the peritubular dentin, which roughens the surface.^18^ These findings are also verified by the SEM observations.

The null hypothesis that there was no difference in root surface roughness and tooth substance loss after chemomechanical challenge between the different root surfaces generated by the hand scaler, ultrasonic tip and erythritol airflow had to be rejected; although no difference in substance loss through chemomechanical challenge was found between the surfaces treated with the hand scaler, the ultrasonic device, and the untreated samples, the dentin surface treated with erythritol-powder airflow exhibited less substance loss through chemomechanical challenge compared to the other pre-treatments. It might be assumed that “airflowing” with erythritol particles leads to a matting effect, partially closing the pores and thus increasing the density of the dentin. As a consequence, the dentin becomes more resistant to toothbrush abrasion thanks to the higher density. Further investigations are warranted in this respect to verify this hypothesis.

Regarding the roughness after chemomechanical challenge, a difference between the different treated surfaces was detected. This study showed a higher surface roughness after chemomechanical challenge on the dentin surface treated with the ultrasonic tip in comparison to the surfaces treated with the hand scaler, airflow with erythritol powder, and the ‘untreated’ surfaces (carborundum grinding papers, no instrumentation), in which no difference was detected. Regarding the differences between roughness after instrumentation and chemomechanical challenge, the following findings were observed. Smoothening through chemomechanical challenge of the surfaces treated with erythritol airflow was observed, as was roughening of those surfaces treated with the ultrasonic device and untreated surfaces. The roughness of the samples treated with the hand scaler did not change through the chemomechanical challenge.

One possible explanation for the greater roughness of the ultrasonically treated samples after chemomechanical challenge is that the bristles of the toothbrush may have entered and deepened the grooves created by the ultrasonic tip, which then led to higher roughness.

## Conclusions

Within the limitations of the present study, it can be concluded that differently pretreated surfaces might have an influence on substance loss and roughness through chemical/chemomechanical challenge. The findings showed that a dentin surface pretreated with erythritol-powder airflow results in less substance loss through chemomechanical challenge than hand-scaled or ultrasonically treated dentin surfaces. Furthermore, the chemomechanical challenge does not eliminate the differences in roughness, in contrast to the hand scaler and erythritol airflow, but increases the surface roughness on surfaces treated ultrasonically.
